# 1-year results of combined half-dose photodynamic therapy and ranibizumab for polypoidal choroidal vasculopathy

**DOI:** 10.1186/s12886-015-0061-8

**Published:** 2015-06-30

**Authors:** Ian Y. Wong, Xuan Shi, Rita Gangwani, Paul Zhao, Lawrence P. Iu, Qing Li, Alex Ng, Xiaoxin Li

**Affiliations:** Department of Ophthalmology, LKS Faculty of Medicine, The University of Hong Kong, Hong Kong, China; Department of Ophthalmology, Peking University People’s Hospital, Beijing, China; Department of Ophthalmology, National University Hospital, Singapore, Singapore

**Keywords:** Age related macular degeneration, Half-dose, Half-fluence, Photodynamic therapy, Polypoidal choroidal vasculopathy, Ranibizumab, Verteporfin

## Abstract

**Background:**

To evaluate the efficacy and safety of half-dose photodynamic therapy (PDT combined with ranibizumab for polypoidal choroidal vasculopathy (PCV). PCV is commonly treated with a combination of anti-vascular endothelial growth factor and standard-dose photodynamic therapy (PDT). Choroidal ischemia and visual loss can be resulted from the standard-dose PDT. Half-dose PDT has proved to produce similar results and safety profile in treating central serous chorioretinopathy. Half-dose PDT may offer an alternative for PCV cases where the damage to choroidal vasculature maybe less. Here, we report the efficacy of treating PCV cases with combination of ranibizumab and half-dose PDT.

**Methods:**

In this prospective, non-comparative, interventional case series, 19 treatment-naive eyes were treated with combined half-dose PDT and ranibizumab. All subjects were followed up for 12 months with measurement of best-corrected visual acuity (BCVA), central foveal thickness (CFT) by optical coherence tomography. Indocyanine green angiogram (ICG) was performed every 3-monthly, and subjects assessed in terms of polyp regression rates, changes in vision and central foveal thickness, need to repeat half-dose PDT. Subgroup analysis was performed based on ICG features.

**Results:**

The mean logMAR BCVA improved from 0.64 at baseline to 0.41 at 12 months. The mean CFT improved from 459.6mum at baseline to 384.2mum at 12 months. The difference between baseline BCVA and CFT and that at 12 months were statistically significant (both *P* = 0.03). Polyp regression rate after one half-dose PDT was 42.1 %. This was 61.5 % in the polyp-only group, while that in the branching-vascular-network (BVN) group was 0 % (*P* = <0.01).

**Conclusion:**

Half-dose PDT combined with intravitreal ranibizumab was able to induce high polyp regression rate in PCV cases that had one single polyp.

## Background

Polypoidal choroidal vasculopathy (PCV) is characterized by polypoidal lesions originating beneath the retinal pigment epithelium (RPE) [1, 2]. It is still being debated whether it is a subtype of wet age-related macular degeneration or an independent pathology [3, 4]. Its reported prevalence is higher in Asian population than Caucasians, and the rate varies between 22.3 % and 54.7 % among Asian countries [5].

Clinically, these polyps appear as protruding elevated orange red lesions. These exist either as isolated polyps, or are associated with a branching vascular network (BVN) [1, 5]. The course of polyps in PCV is variable, and can be associated with serous exudation and hemorrhage which may lead to RPE detachments. At times, it also gives rise to subretinal fluid (SRF) with detachments of neurosensory retina [5, 6].

The recommended treatment for PCV is either combination of standard-fluence verteporfin PDT and intravitreal injections of anti-vascular endothelial growth factors (anti-VEGFs) at monthly intervals, or a standard-fluence verteporfin PDT [1, 7]. The mechanism of action of PDT is postulated to be short-term choriocapillaris hypoperfusion and long-term choroidal vascular remodeling, leading to reduction in choroidal congestion, vascular hyperpermeability, and extravascular leakage [8].

Despite the demonstrated efficacy of PDT with full-dose verteporfin in inducing polyp regression, potential adverse events exists, such as secondary RPE changes at the site of PDT laser application, which is the result of hypoxic damage caused by choriocapillaris occlusion [9]. Some have demonstrated transient reduction in macular function and even reduction in choroidal circulation following PDT [10]. Choroidal neovascularization (CNV) can develop after PDT for other retinal conditions such as central serous chorioretinopathy (CSC) due to choroidal ischaemia.

To reduce the risks of PDT, the intensity of treatment can be reduced, either by reducing the fluence of PDT or by reducing the dose of verteporfin. Recently, there have been reports of success with half-fluence PDT in the treatment of PCV combined with anti-VEGF injections [12–14]. In the case of CSC, half-dose PDT was tried and good results were shown [11]. Half-dose PDT was also found to be more effective than half-fluence PDT in the treatment of CSC [15]. However, we are not aware of any studies looking into the efficacy of half-dose PDT in the treatment of PCV. The purpose of this study was to determine the efficacy of half-dose PDT for the treatment of PCV, in combination with intravitreal ranibizumab.

## Methods

This was a prospective, consecutive, open-label, non-comparative interventional study, carried out at two sites: the University of Hong Kong, and the Peking University People’s Hospital. This study adhered to the Declaration of Helsinki and ethics approval was obtained from the Institutional Review Boards of the two sites (Institutional Review Board of the University of Hong Kong/Hospital Authority Hong Kong West Cluster reference number UW12-207). Same protocol was adopted at the two sites, and the study was performed simultaneously. Informed consent was obtained from all subjects.

The main inclusion criteria were 1) treatment naïve PCV as characterized by the presence of polyps or branching vascular networks (BVN) on indocyanine green angiogram (ICG), 2) age over 50 years, 3) best-corrected visual acuity (BCVA) of 0.30 or worse (LogMAR, hereafter), and 4) a greatest linear dimension (GLD) of 5400 μm or less. Exclusion criteria were 1) prior treatment with either intravitreal injections of anti-VEGF of any kind or verteporfin PDT, 2) the presence of vitreous hemorrhage, 3) extensive subretinal hemorrhage preventing proper imaging/PDT from being performed, 4) an area of scar tissue accounting for 50 % or more of the lesion, and 5) presence of other retinal diseases such as diabetic retinopathy.

All consecutive eligible subjects were recruited from 1 Sep 2012 to 30 Jun 2013, and underwent comprehensive baseline ophthalmic examination and at monthly intervals for 12 months. These included BCVA (logMAR, hereafter), macular examination with a 90-D lens, fundus photography, and optical coherence tomography (OCT) (Heidelberg Spectralis; Heidelberg Engineering, Heidelberg, Germany). Fluorescein angiogram (FA) and indocyanine green angiogram (ICG) were performed using a scanning laser ophthalmoscope (Heidelberg Retinal Angiography; Heidelberg Engineering, Heidelberg, Germany) at baseline and every 3 months.

All subjects received a combination of intravitreal ranibizumab (Lucentis; Novartis AG, Basel, Switzerland) and half-dose verteporfin (Visudyne; Novartis AG, Basel, Switzerland) PDT after enrollment as initial treatment. The half-dose PDT was given within 7 days after intravitreal ranibizumab. Ranibizumab (0.5 mg/0.05 ml) was injected 3.5 mm post-limbus using a 30-gauge needle under aseptic condition. Half-dose PDT was performed using half of the normal dose of verteporfin (3 mg/m^2^ verteporfin). Verteporfin was infused over 10 min, followed by delivery of laser at 689 nm at 15 min from the commencement of infusion. The laser spot size was determined by adding 1000 μm to the entire PCV lesion, including any polyps and/or BVN as seen on ICG. A total light energy of 50 J/cm^2^ over 83 s was delivered.

Subjects were reassessed monthly. If there was disease activity on OCT, defined as presence of any subretinal and/or intraretinal fluid, ranibizumab would be given. FA and ICG were repeated every 3 months, if 1) no polyps or BVN were seen on ICG, and no disease activity on OCT and FA (defined as presence of FA leakage), no retreatment would be given; 2) if polyps were seen and there was FA / OCT disease activity, half-dose PDT and ranibizumab would be repeated; 3) if only BVN was seen without polyps, and there was FA/OCT disease activity, only ranibizumab would be given; and 4) if only BVN was seen without polyps, and there was no FA/OCT disease activity, no re-treatment would be given.

Primary outcomes measured were the change in BCVA and the rate of polyp/BVN regression after half-dose PDT during the study period. Secondary outcomes included change in central foveal thickness (CFT) on OCT and the number of half-dose PDT sessions required to induce polyp/BVN regression. Subgroup analysis was performed between cases that had polyp-only and cases with BVN on ICG.

Microsoft Excel (Microsoft Excel 2011) was used for data collection; SPSS was used for statistical analysis (SPSS Version 19). Chi-square test was used to evaluate the differences between the proportions of subjects achieving the measurement endpoints. Fisher’s exact test was used when the expected frequency of a cell in a table was less than 5. Two sample t-tests were used to measure differences in the continuous variables, such as age and CFT, between the treatment groups. A probability level of <0.05 was used to measure statistical significance. All of the tests were two-sided.

## Results

Demographics and summary of the responses are shown in the Table 1. Nineteen eyes of 19 subjects were recruited (13 at the University of Hong Kong, 6 at the Peking University People’s Hospital). Ten of them were male (52.6 %), and the mean age was 64.8 ± 15.2 years (range 22 to 88 years). Thirteen eyes (67.4 %) had polyps only, 7 of which had only 1 polyp, while 6 had more than 1 polyp (range 2 to 5). Six eyes (32.6 %) had both polyps and BVN, and amongst the cases with BVN, all had more than 1 polyp.

**Table 1:** Background characteristics and treatment outcomes of the subjects

Subject	Age/Sex	Baseline ICG features	Number of Half-dose PDT	BCVA (logMAR)				Number of ranibizumab injections	ICG findings at 12-month		Complications
				Baseline	3-month	6-month	12-month		Polyp	BVN	
1	60/F	1 polyp	1	0.7	0.4	0.3	0.3	3	Regressed	/	Nil
2	60/F	1 polyp	1	1.0	0.55	0.3	0.3	3	Regressed	/	Nil
3	77/M	1 polyp	1	0.3	0.3	0.2	0.2	2	Regressed	/	Nil
4	68/F	1 polyp	1	0.3	0.3	0.4	0.3	4	Regressed	/	Nil
5	66/M	1 polyp	1	0.0	0.1	0.0	0.1	1	Regressed	/	Nil
6	71/M	1 polyp	1	0.1	0.1	0.1	0.1	1	Regressed	/	Nil
7	55/M	1 polyp	1	1.3	0.4	0.4	0.4	2	Regressed	/	Nil
8	63/M	2 polyps	1	0.7	0.4	0.4	0.3	1	Regressed	/	Nil
9	52/F	2 polyps	2	1.0	0.7	0.7	0.6	2	Regressed	/	Nil
10	88/M	2 polyps	2	0.55	0.4	0.4	0.4	2	Regressed	/	Nil
11	78/M	2 polyps	2	0.7	0.4	0.3	0.4	2	Regressed	/	Nil
12	75/F	2 polyps	2	0.55	0.3	0.4	0.3	1	Regressed	/	Nil
13	60/F	5 polyps	2	1.0	0.7	1.0	1.0	7	Regressed	/	Nil
14	69/M	BVN + 2 polyps	2	0.4	0.4	0.55	0.4	2	Regressed	Persisted	Nil
15	72/F	BVN + 2 polyps	2	0.7	0.7	0.55	0.55	2	Regressed	Persisted	Nil
16	55/F	BVN + 2 polyps	3	1.3	1.0	1.0	1.0	5	Regressed	Persisted	Nil
17	84/F	BVN + 2 polyps	2	0.3	0.3	0.4	0.3	1	Regressed	Persisted	Nil
18	76/M	BVN + 3 polyps	2	0.55	0.4	0.3	0.3	2	Regressed	Persisted	Nil
19	81/M	BVN + 5 polyps	2	0.7	0.4	0.55	0.55	2	Regressed	Persisted	Nil

**Fig. 1:**
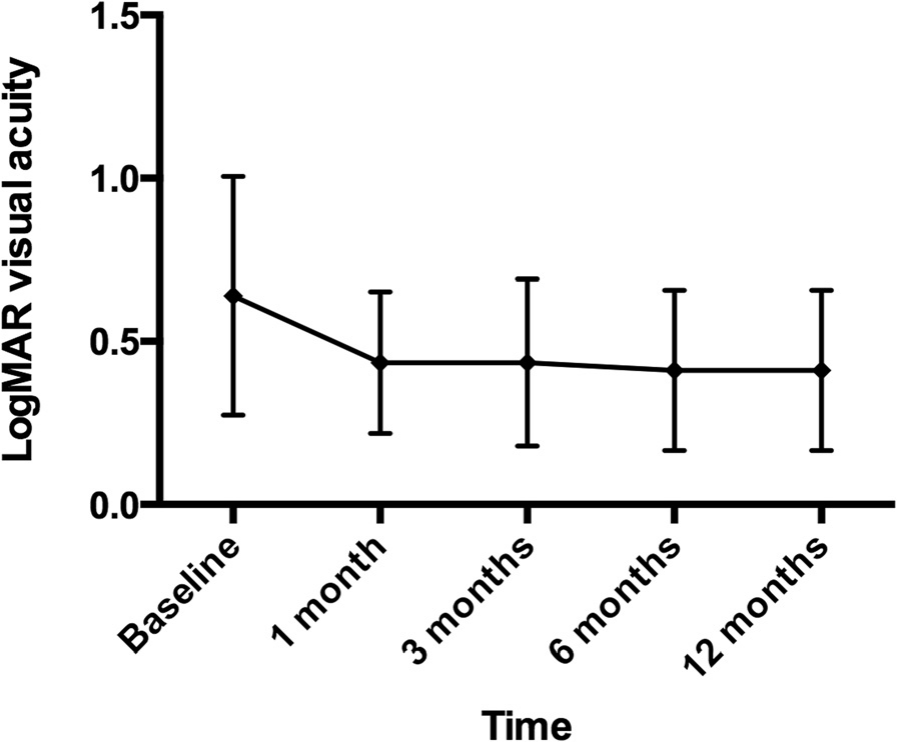
The changes in mean logMAR best-corrected visual acuity over time

**Fig. 2:**
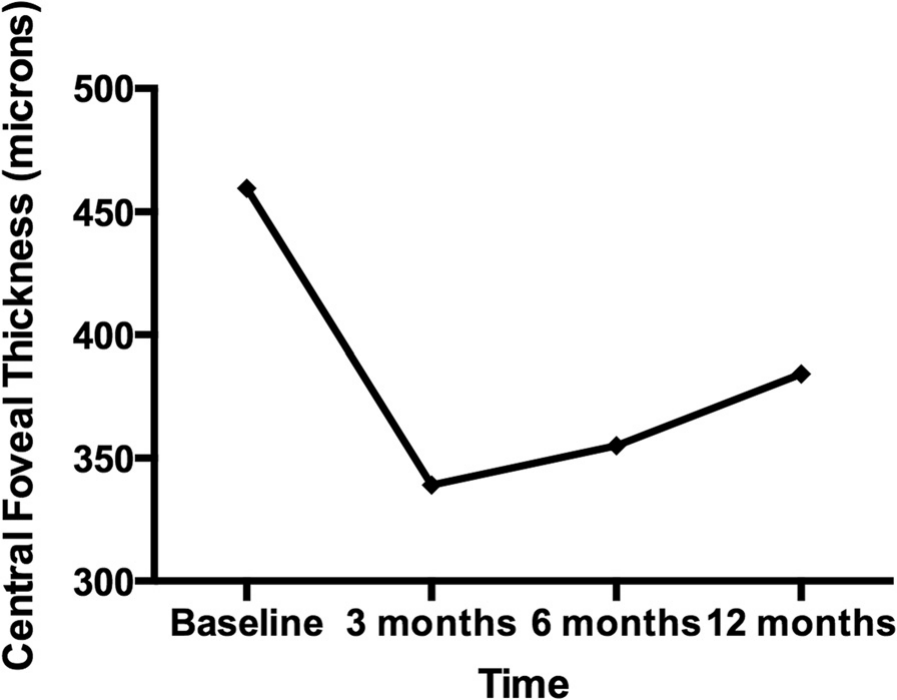
The changes in mean central foveal thickness on optical coherence tomography over time

The overall mean BCVA were 0.64 ± 0.37 at baseline, 0.43 ± 0.22 at 3 months, 0.43 ± 0.26 at 6 months, and 0.41 ± 0.25 at 12 months. The mean CFT were 459.6 ± 167.3 μm at baseline, 339.0 ± 175.8 μm at 3 months, 355.0 ± 181.1 μm at 6 months, and 384.2 ± 194.9 μm at 12 months (Figs. 1 and 2). The difference between baseline BCVA and CFT and that at 12 months were statistically significant (*P* = 0.03, *P* = <0.01 respectively).

Visual acuity in the polyp-only group was better than the BVN group in all time points, but the difference did not differ statistically (*P* = 0.19 at baseline, *P* = 0.90 at 3 months, *P* = 0.28 at 6 months, *P* = 0.98 at 12 months). The mean number of ranibizumab injections was 2.37 ± 1.5 (range 1 to 7) throughout the 12-month period.

**Fig. 3:**
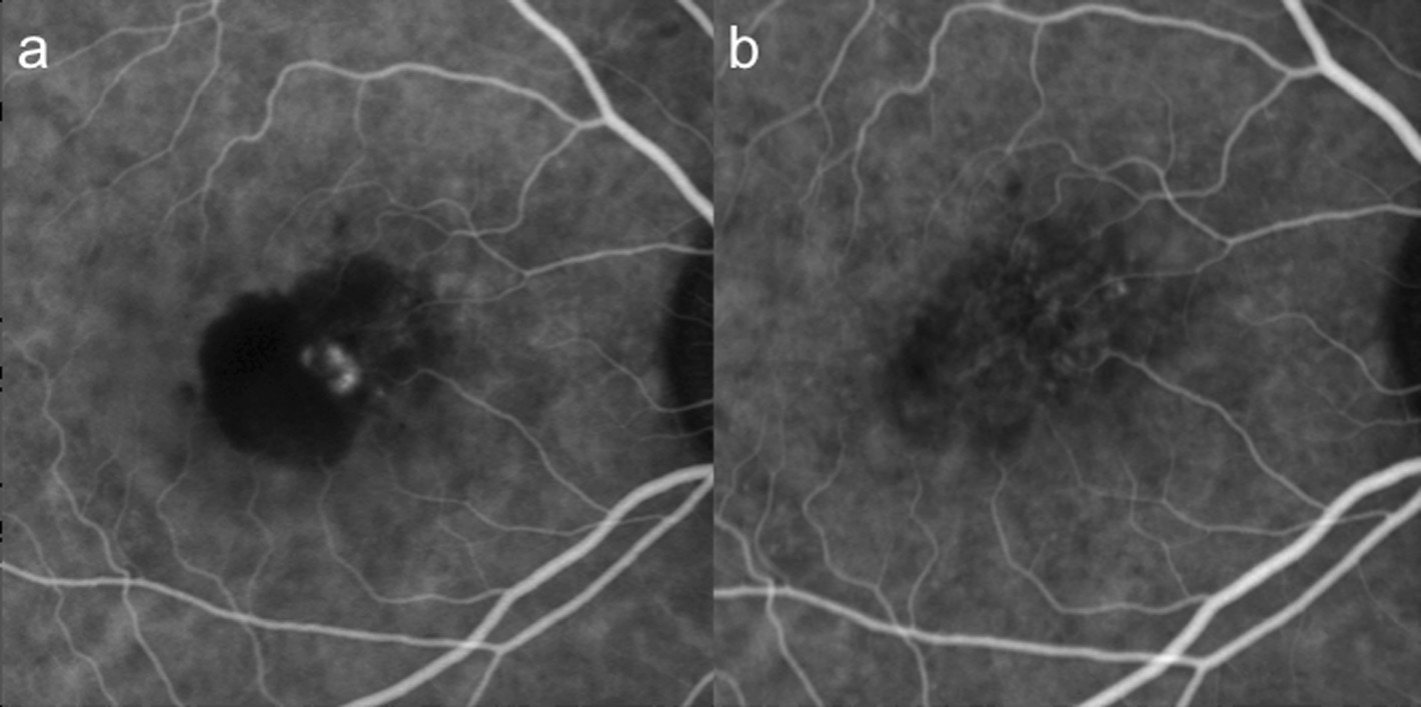
This was the indocyanine green angiogram of subject 6. Baseline logMAR visual acuity was 0.1, and remained at 0.1 throughout. There was only 1 single polyp, which regressed after only one session of half-dose photodynamic therapy and one injection of ranibizumab. **a** Showed the appearance of the polyp at baseline on ICG; and **b** the appearance at 3 months

**Fig. 4:**
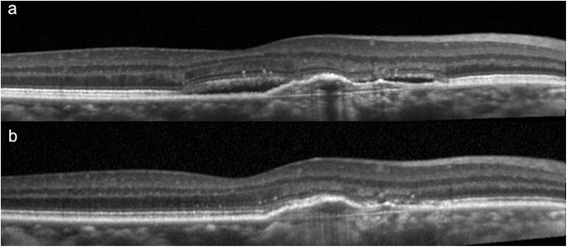
This was the optical coherence tomography of subject 6 at (**a**) baseline, and (**b**) at 3 months. This showed the complete resolution of subretinal fluid

With 1 session of half-dose PDT combined with 1 injection of intravitreal ranibizumab, complete polyp regression (Figs. 3 and 4) was achieved in 8 out of 19 eyes (42.1 %). Out of these 8 cases, 7 of which (87.5 %) had only 1 polyp at baseline, and the remaining one case had 2 polyps.

For the remaining 11 eyes (57.9 %), after one session of half-dose PDT, in 8 eyes, although polyps persisted on ICG, the polyps size reduced. In the remaining 3 eyes, the polyps did not change after one session of half-dose PDT. All the BVN did not change after one session of half-dose PDT.

Polyp regression rate after one single half-dose PDT session was 61.5 % in the polyp-only group, while that in the BVN group was 0 % (*P* = <0.01). Of particular interest, the polyp regression rate was 100 % for those that had only 1 polyp and no BVN at baseline (7 out of 7 eyes).

Re-treatment rate of half-dose PDT in the polyp-only group (*n* = 13) was 38.5 %, and the mean number of half-dose PDT sessions was 1.38 (range 1 to 2). That in the BVN group was 100 % and the mean number of sessions was 2.17 (range 2 to 3) (*P* = 0.04).

The 8 cases that had polyp regressed after one session of half-dose PDT required no further treatments with half-dose PDT. Of the 6 eyes with BVN, none showed regression throughout the 12-month period. However, after a mean of 2.17 (range 2 to 3) sessions of half-dose PDT, no disease activity was seen despite persistence of the BVN on ICG.

Retreatment with ranibizumab was required in both groups. Overall mean number of ranibizumab in the 12-month period was 2.37 ± 1.5 (range 1 to 7). That in the polyp-only group was 2.38; while that in the BVN group was 2.33, and the difference was not statistically significant (*P* = 0.70).

No significant adverse events occurred during the 12-month period. No loss of visual acuity of more than 3 lines in any of the eyes. No systemic side effects were observed.

## Discussion

PDT is an established treatment modality for PCV [1, 27]. The mechanism of action involves a vaso-occlusive effect. When PDT activates the photosensitizer verteporfin at the laser-applied area, it induces vascular thrombosis, reduced perfusion and PCV regression [8, 9]. A high polyp regression rate of 82 to 95 % with PDT monotherapy was reported [8, 16, 17]. The standard PDT dosage had been shown in studies to cause choroidal ischaemia, RPE atrophy, secondary CNV [18] and fibrous scarring [18] that limit the visual gain despite PCV regression. In addition, persistence of BVN with PCV recurrence is not uncommon after treatment [9]. Repeated PDTs would produce additive adverse effects [19]. As a result, the long-term efficacy of PDT is often disappointing. In a prospective study of 65 eyes, the mean BCVA decreased an average of 0.21 logMAR units at 5 years after initial PDT. In another retrospective study of 43 eyes [20], BCVA decreased to below baseline in all eyes after 3 years. The authors attributed the unfavorable long-term outcomes to foveal atrophy [20], PCV recurrence [20, 21] and CNV development [20]. Therefore, methods to reduce the adverse effects of PDT are necessary. One approach is to minimize the treatment intensity by reducing the fluence; the other is to reduce the dose of verteporfin.

Initial studies showed favorable outcomes with reduced-fluence PDT in treating PCV [12, 14, 22, 23]. Yamashita et al. demonstrated that, in a prospective study of 38 eyes treated with reduced-fluence PDT, the mean BCVA improved from 0.43 to 0.29 logMAR units at 2 years [22]. The mean treatment sessions were 1.9 only. The BCVA remained stable or improved in 95 %. Polyp regressed in 92 % at 3 months. When reduced-fluence PDT was used in combination with intravitreal ranibizumab, Ricci et al. showed a significant improvement of BCVA from 0.45 to 0.29 logMAR units at 1 year [23]. The BCVA remained stable or improved in 95 %. Polyp regressed in 94 %. Sakurai et al. showed a significant improvement of BCVA from 0.55 to 0.38 logMAR units at 1 year, and fewer ranibizumab treatments were required [12]. Sagong et al. showed in a prospective study that combining intravitreal bevacizumab and reduced-fluence PDT, the BCVA improved from 0.76 to 0.46 logMAR at 1 year [14].

Concerning the problems with reduced-fluence PDT, a high rate of BVN persistence of 84 % at 3 months [22] and 65 % at 1 year were reported [23]. Subretinal hemorrhage within 3 months after PDT (13 %, Yamashita et al. [22]), persistent mild choriocapillary non-perfusion (18.8 %, Sagong et al. [14]) and persistent mild-to-moderate choriocapillary non-perfusion (20 %, Yoshida et al. [13]) had also been reported. Therefore, despite good initial visual outcome, the risk of polyp recurrence and choroidal ischaemia still exist even when the fluence had been reduced.

Half-dose PDT has been widely used for the treatment of CSC [11, 24] and is considered to have fewer side effects than standard-dose PDT [24]. Improvement in visual acuity [11, 24], contrast sensitivity, microperimetric retinal sensitivity and retinal function on multifocal electroretinography [25] had been demonstrated following treatment of CSC with half-dose PDT. Nicolo et al. evaluated the efficacy and safety of half-dose versus half-fluence PDT and showed that half-dose PDT induced more rapid reabsorption of fluid and more lasting effect with equal safety [15]. Since both CSC and PCV have similar underlying pathophysiology of choroidal hyperpermeability [26, 27], half-dose PDT might also be more effective than half-fluence PDT in PCV treatment. To the best of our knowledge, this is the first study to investigate the efficacy of half-dose PDT and ranibizumab in PCV.

This study showed that half-dose PDT combined with ranibizumab was highly successful in treating single small polyp PCVs, but it appeared to be less effective if there were BVN and/or multiple polyps were present. In patients who had a single small polyp, we observed polyp regression or disease activity resolution for at least 9 months after treatment. For cases with more than 1 polyp, although a single session of half-dose PDT was not able to induce polyp regression in majority of the cases, repeated treatment achieved polyp regression at a later time point. Although the current regimen was not able to cause BVN regression, it appeared that it was able to suppress the disease activity associated with the BVN. Our result suggested that the number of polyps and the presence of BVN were important prognostic factors that determined the success rate of combined half-dose PDT and ranibizumab.

Another point to note is that subjects with polyp-only or had BVN did not behave differently in terms of the visual acuity improvement and number of ranibizumab injections required. This was probably because of the efficacy of the half-dose PDT. For the polyp-only cases it was rather straightforward in that regression of the lesions led to clinical improvement; in subjects with BVN, one explanation is that even though the half-dose PDT did not lead to regression of the BVN, it induced stability of the lesion. That was probably why clinically these cases behaved well despite the persistence of the lesion on ICG. Whether the endpoint of PDT (be it half-dose, half-fluence, or standard) is complete regression of the lesions or not requires further investigation.

The limitations of this study included small sample size, the lack of comparison with standard-dose PDT, the lack of comparion with half-fluence PDT, and the heterogeneity in lesion types recruited. However, the heterogeneity of subjects may be a reflection of the high variability of the disease itself. Our findings suggested that half-dose PDT is highly successful for selected cases of PCV. Future controlled studies are warranted to determine the long-term efficacy of half-dose PDT, to identify the optimal lesion type for treatment, and to compare the efficacy and safety with standard-dose PDT.

## Conclusion

Half-dose PDT combined with intravitreal ranibizumab was able to induce high polyp regression rate in PCV cases that had one single polyp.
